# (Dys)function Follows Form: Nucleic Acid Structure, Repeat Expansion, and Disease Pathology in *FMR1* Disorders

**DOI:** 10.3390/ijms22179167

**Published:** 2021-08-25

**Authors:** Xiaonan Zhao, Karen Usdin

**Affiliations:** Laboratory of Cell and Molecular Biology, National Institute of Diabetes, Digestive and Kidney Diseases, National Institutes of Health, Bethesda, MD 20892, USA

**Keywords:** fragile X-associated primary ovarian insufficiency (FXPOI), fragile X-associated tremor/ataxia syndrome (FXTAS), fragile X syndrome (FXS), repeat instability, repeat expansion, chromosome fragility, RNA gain-of-function, repeat-associated non-AUG (RAN) translation, repeat-mediated gene silencing

## Abstract

Fragile X-related disorders (FXDs), also known as *FMR1* disorders, are examples of repeat expansion diseases (REDs), clinical conditions that arise from an increase in the number of repeats in a disease-specific microsatellite. In the case of FXDs, the repeat unit is CGG/CCG and the repeat tract is located in the 5′ UTR of the X-linked *FMR1* gene. Expansion can result in neurodegeneration, ovarian dysfunction, or intellectual disability depending on the number of repeats in the expanded allele. A growing body of evidence suggests that the mutational mechanisms responsible for many REDs share several common features. It is also increasingly apparent that in some of these diseases the pathologic consequences of expansion may arise in similar ways. It has long been known that many of the disease-associated repeats form unusual DNA and RNA structures. This review will focus on what is known about these structures, the proteins with which they interact, and how they may be related to the causative mutation and disease pathology in the *FMR1* disorders.

## 1. Introduction

Repeat expansion diseases (REDs) are a group of human diseases caused by the presence of a large number of repeats in a microsatellite or short tandem repeat (STR) [1]. Unlike the microsatellite instability caused by a mismatch repair (MMR) deficiency that affects STRs genome-wide, each of these diseases results from expansion at a single disease-specific locus. While contractions of the repeat are occasionally seen, expansions predominate in both somatic and germline cells. The propensity to expand becomes apparent when the repeat number exceeds a certain critical threshold, with expansions increasing in frequency as the repeat number increases. These expansions occur in both intergenerational transmission and in the somatic cells during the lifetime of the individual. In general, for many of the diseases that are not congenital, the age at onset decreases and disease severity or disease penetrance increase with increasing repeat number [1]. As will be discussed in more detail later in this review, the characteristic features and genetic requirements for expansion in many of these diseases suggest that they may arise in similar ways. Furthermore, the pathology in many of these diseases may also arise from similar consequences of the expansion process.

More than 40 REDs have been identified to date, including Huntington’s disease (HD), myotonic dystrophy type 1 (DM1), *C9orf72*-associated amyotrophic lateral sclerosis/frontotemporal dementia (ALS/FTD), and the *FMR1* disorders, also known as the fragile X (FX)-related disorders (FXDs). In the case of the FXDs, the repeat unit is CGG/CCG and the repeat tract is located in the 5′ UTR of *FMR1*, a gene located on the long arm of the X chromosome (reviewed in [2]). Normal alleles have 15–45 repeats, with alleles with ~30 repeats being the most common. In this context the repeat tract is thought to play a role in the regulation of synthesis of the *FMR1* gene product, FMRP, via the production of a protein generated from an upstream open reading frame using a near-cognate AUG codon [3]. Normal alleles are relatively stable. However, larger alleles tend to expand both in germline [4] and somatic cells [5]. Most of the historical focus has been on germline expansion, and while somatic expansion does play a role in other REDs [6,7], its role in the FXDs is unknown. Expanded repeats have paradoxical effects on expression of the *FMR1* gene, with alleles with 55–200 repeats (known as premutation (PM) alleles [2,8]) being hyper-expressed, and alleles with >200 repeats (known as full mutation (FM) alleles [9,10]) being epigenetically silenced. The net result is that females with PM alleles are at risk of a form of female infertility known as fragile X-associated primary ovarian insufficiency (FXPOI), and both PM males and females are at risk for a neurodegenerative condition known as fragile X-associated tremor/ataxia syndrome (FXTAS). The PM is seen in 1:200 females and 1:400 males [11]. Penetrance for FXTAS increases with age and repeat number, with >60% of male PM carriers showing symptoms by age 70, as compared to ~16% of females [11]. FXPOI affects ~20–30% of PM carriers [12] and there is a non-linear relationship between repeat number and FXPOI risk that is not well understood [13,14]. Cells from PM carriers show splicing abnormalities [15], lamin A/C dysregulation [16], mitochondrial disfunction, and the presence of intranuclear inclusions in the brain and ovary [17,18]. Female PM carriers are at risk of transmitting FM alleles to their children, with the risk of doing so being related to their repeat number, the number of AGG interruptions seen at the 5′ end of the repeat tract, and maternal age [19]. The risk of maternal transmission of a FM allele approaches 100% when the repeat number exceeds 90, irrespective of age or interruptions [19]. In contrast, male PM carriers do not transmit FM alleles, likely due to the tendency of long repeat tracts to contract in sperm [20]. FM alleles are seen at a frequency of ~1 in 2000 to 1 in 7000 in the general population, with a variation in prevalence seen in different populations [21]. Most males who inherit FM alleles have fragile X syndrome (FXS), the most common monogenic cause of intellectual disability and autism [22]. Females tend to be less severely affected due to the protective effect of their second X chromosome. Silencing of the FM allele results in the loss of FMRP, a multi-functional protein best known for its role in negatively regulating the translation of genes important for learning and memory [23]. FM alleles are also associated with a folate-sensitive fragile site, a gap or constriction of the chromosome, coincident with the repeat [10]. Female FM fetuses also show a high frequency loss of the affected X chromosome, resulting in Turner syndrome [24].

**Figure 1:**
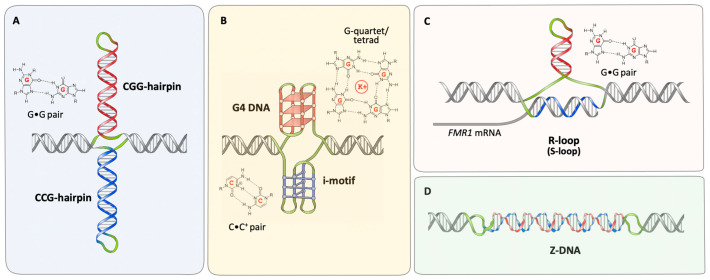
Generic representation of the types of DNA and RNA structures formed by FX repeats. Structures shown include hairpins formed by each strand of the repeat (**A**), a quadruplex or G4 DNA structure and an i-motif structure (**B**), an R-loop with associated hairpin formed by the non-template strand resulting in an S-loop (**C**) and Z-DNA (**D**). The CGG strand is shown in red and the CCG strand in blue. Unpaired loops regions are shown in green and the non-repetitive flanking DNA is shown in grey. Note that in addition to unpaired loop bases, some of these structures also contain non-Watson Crick base pairs or mismatches. The structures of the constituent non-canonical base interactions are shown alongside each structure.

As with other expansion-prone repeats, the CGG/CCG repeats responsible for the *FMR1* disorders form a variety of nucleic acid secondary structures (Figure 1). These structures have the potential to interfere with many biological processes. As such, they have the potential not only to cause the mutation responsible for the FXDs, but they may also be responsible for some of the pathological consequences of the mutation. Interestingly, many targets of FMRP form G4 structures to which the protein binds [25], and FMRP has also been implicated in R-loop processing [26], thus representing other ways that non-canonical nucleic acid structures and proteins intersect in these disorders. However, in this review we will focus primarily on what is known about the DNA and RNA structures formed by the FX repeats themselves and their biological effects in the context of both expansion and disease pathology in the *FMR1* disorders.

## 2. Secondary Structures Formed by FX Repeats

Like the repeats responsible for many of the other REDs, individual DNA strands of the FX repeat can form stable hairpins containing a mixture of Watson–Crick and non-Watson–Crick base pairs or mismatches [27,28,29,30,31,32]. CGG-DNA hairpins are the most stable of the hairpins formed by different trinucleotide repeats, with a (CGG)_15_ hairpin having a Tm of 75 °C in physiologically reasonable buffers [33]. In contrast, similarly sized CCG hairpins have a Tm of 30–37 °C depending on pH, and are less stable than CGG, CTG, and CAG repeats [33]. While similar experiments have not been performed for CGG and CCG repeats, evidence from cleavage by zinc finger nucleases specific for CAG and CTG repeats provides evidence for the formation of such hairpins in mammalian cells [34]. In principle, hairpin formation by both strands of the repeat could result in a cruciform-like structure, as illustrated in Figure 1A. CGG repeats also form stable hairpins in RNA [35,36,37]. In addition to hairpins, the formation of intramolecular and intermolecular G4 quadruplex structures by both CGG repeat-containing DNA and RNA have been reported in some studies [27,38,39,40,41,42,43,44,45] (Figure 1B). These structures are sometimes overlooked because CGG hairpins form readily and once formed are very stable, whilst the G4 structures are only seen in the presence of K^+^ [27]. Nonetheless, once formed these structures are stable at temperatures of >85 °C with physiologically reasonable K^+^ concentrations [27]. The CCG strand of the repeat has also been shown to form a variety of intramolecular and intermolecular four-stranded structures, including i-motif structures containing intercalated C•C^+^ base pairs [46,47,48] as illustrated in Figure 1B.

In addition to intrastrand DNA and RNA structures, the 5′ end of the *FMR1* gene forms a stable R-loop in vivo, as illustrated in Figure 1C [49,50,51,52]. In these structures, the G-rich transcript forms a hybrid with the C-rich template strand, likely during transcription. This results in a three-stranded structure involving an RNA:DNA hybrid and a displaced DNA strand. The *FMR1* R-loop extends well into the 5′ and 3′ flanking regions [49,51], regions that also have a strong GC skew [53]. Non-denaturing bisulfite mapping shows that most of the cytosines on the non-template strand are resistant to bisulfite modification [49], consistent with the formation of intrastrand folded structures by the non-template strand. An R-loop containing a non-template-strand hairpin, sometimes referred to as an S-loop (for slipped hairpin R-loops), is illustrated in Figure 1C, but an R-loop with a G4 structure, a G-loop, is also possible. In either case the occasional modified cytosines seen on the bisulfite-treated non-template strand [49] would correspond to bases in the loops of these structures. Structures formed by the non-template strand may in turn help stabilize the R-loop [54]. Since the CGG/CCG repeats at the *FMR1* locus are bidirectionally transcribed, they can also form double R-loops [55]. In addition to these inter- and intra-strand structures, there is evidence that even the CGG•CCG duplex is atypical, adopting a left-handed Z-DNA conformation as illustrated in Figure 1D [56].

## 3. Repeat Expansion

One important clue to the process that causes repeat expansion in the REDs has emerged from recent genome-wide association studies (GWAS) in different RED patient cohorts. These studies have implicated the MMR proteins MSH3, MLH1, and MLH3 as important modifiers of somatic expansion risk and/or age at onset/disease severity in many REDs [6,7,57,58,59,60,61,62,63]. MSH3 forms a heterodimer with MSH2 in the MutSβ complex, one of the two mismatch recognition complexes involved in MMR in mammals, while MLH1 and MLH3 form the heterodimer MutLγ, a complex that acts downstream of MutSβ in the MMR pathway [64]. Notably, single nucleotide polymorphisms associated with increased MSH3 expression are associated with increased somatic expansion in an HD patient cohort [7], suggesting that, unlike the microsatellite instability associated with certain cancers, functional MMR proteins are required for expansion. A requirement of these same proteins for repeat expansion is seen in a mouse model of FXDs as well as other mouse models of REDs (reviewed in [65,66]). A role for MMR in repeat expansion is consistent with the fact that many of the unusual structures formed by the repeats contain mismatches or regions of single-strandedness that can be bound by MutSβ and the related protein MutSα, a heterodimer of MSH2 and MSH6 [64,67]. While GWAS studies of factors that affect germline expansion risk have not yet been performed for REDs, in the FXD mouse model it is known that the same factors that affect somatic expansion risk also affect germline expansion risk (reviewed in [65]).

However, how the MMR substrates arise is unclear. It may be that they form during strand slippage or strand displacement during replication or repair. Since expansion in many REDs can occur in non-dividing cells like oocytes and neurons [19,68], repair may be a more likely source of these substrates, at least in disease-relevant cell types. One model for expansion invokes a role of base excision repair (BER) of 7,8-dihydro-8-oxoguanine (8-oxoG), the most common oxidation product in DNA, with strand slippage or strand displacement during BER generating hairpin loop-outs that are bound by the MutS proteins [69]. Hairpin formation may trigger multiple rounds of BER since guanines in the loop of hairpins are susceptible to DNA damage and are less likely to be repaired [70]. A role for BER would be consistent with the fact that loss of the 7,8-dihydro-8-oxoguanine glycosylase (OGG1) leads to reduced expansion in the liver (but not in the brain or gametes) of an HD mouse model [69]. Loss of NEIL1, the other major DNA glycosylase able to remove 8-oxoG, also led to a decline in expansion in HD mouse brain [71]. GWAS studies in other REDs have not as yet identified a role for BER proteins in the expansion process [6,7,57,58,59,60,61,62,63]. However, this does not definitively rule out a role for BER. A role for oxidative damage in repeat expansion is supported by the observation that oxidizing agents increase repeat expansion in a mouse model of FXDs [72] and in cell models of HD [73]. However, antioxidants have no effect on an FXD mouse cell model (Miller and Usdin, unpublished observations), and only a modest effect on repeat expansions in HD mouse models [74,75]. Thus, spontaneous oxidative damage may not be a major contributor to expansion under normal circumstances. 

**Figure 2:**
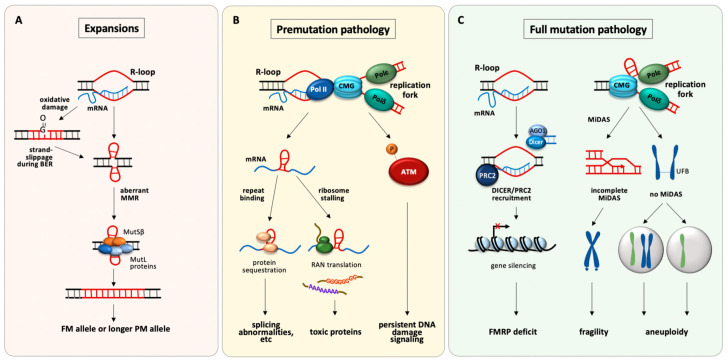
Models for the roles of non-canonical DNA and RNA structures in the etiology of the repeat expansion mutation and the resultant pathology seen in individuals with PM and FM alleles. (**A**) R-loops formed during transcription contain a region of single-stranded DNA that would be prone to oxidative damage. Base excision repair of such damage could generate loop-outs by strand-slippage or strand-displacement during repair synthesis [71,77,78]. R-loops may also facilitate the direct formation of loop-outs, first by the unpaired non-template strand when the template strand is involved in the RNA:DNA hybrid, and subsequently by the template strand after the R-loop is resolved. The loop-outs are bound by mismatch repair factors like MutSβ and MutLγ [79,80,81,82] and are processed via a DSB [83] to generate expansions. (**B**) CGG-hairpins in the *FMR1* transcript can bind and sequester proteins [84,85] or trigger RAN translation of toxic proteins [86,87]. Persistent R-loops, perhaps exacerbated by replication-transcription collisions may result in DSBs that cause persistent DNA damage signaling [49]. (**C**) R-loop formation allows the recruitment of PRC2 to the *FMR1* gene [88]. DICER complexes associated with dsRNA produced from the *FMR1* locus [36] may also contribute to silencing by facilitating recruitment of SUV39H [89]. Secondary structures may cause stalling of the replication fork that triggers MiDAS [90]. Failure to complete MiDAS results in chromosome fragility, while failure to initiate MiDAS results in the formation of UFBs and ultimately the gain or loss of the affected X chromosome [90].

Furthermore, expansions in human PM carriers require transcription of the *FMR1* gene or at least for the allele to be in a region of transcriptionally competent chromatin [76]. Canonical BER has no such strict transcriptional requirement, although it is possible that transcription provides the opportunity for secondary structures to form that in turn would be predisposed to oxidative damage [70]. An alternative source of MMR substrates may be transcription itself, which can result in the formation of an S-loop as illustrated in Figure 1C. The S-loop may be the MMR target. It is also possible that resolution of the R-loop would then leave the template strand unable to bind its complementary strand and since the CCG-rich strand can also form hairpins, this could result in the cruciform-like double loop-out structure shown in Figure 1A and Figure 2A that could also be a target for MMR.

Work on a mouse model of the FXDs shows a dependence on both MutSβ and MutLγ for repeat expansion [79,80,81,82,91], consistent with GWAS of REDs. However, other genetic modifiers of expansion risk in this mouse model suggest that the MMR protein-dependent expansion pathway differs in key ways from canonical MMR. For example, in addition to MutSβ, MutSα also plays an important role in expansion [64], as do MutLα and MutLβ, two other MLH1 containing complexes found in mammals [80]. MutSβ and MutSα are not known to act together in MMR. Neither are MutLγ and MutLα, while the contribution of MutLβ to MMR is unclear. Furthermore, DNA ligase IV, which is required for non-homologous end-joining (NHEJ), a form of double-strand break (DSB) repair, protects against expansion in a mouse model of FXDs [83]. This suggests that expansion involves a DSB intermediate. It may be that a DSB results from cleavage of a double loop-out by MutLγ which normally cuts the strand opposite a mismatch [92]. However, the details of this process and the downstream events that result in the generation of an expansion are still unknown.

## 4. Consequences of Repeat Expansion

### 4.1. Pathology in PM Carriers

Most work on PM pathology has focused on FXTAS rather than FXPOI. While relatively little is known about which cells are most vulnerable in these disorders, it could be that similar mechanisms act to reduce cell viability in both cases. The fact that FM carriers who make little, if any, *FMR1* mRNA and FMRP, do not show FXTAS or FXPOI symptoms suggests that the CGG-repeat-containing RNA produced from PM alleles is responsible, rather than any decline in the amount of FMRP. An RNA-based pathology is supported by the demonstration that ectopic expression of the CGG-tract causes reduced cell viability [72,93,94,95,96,97], the production of inclusions [94,98,99], disruption of the nuclear lamin A/C architecture in neuronal cell lines [16], and neurodegeneration in both flies [94] and mice [96]. It also alters the ovarian response to gonadotropins and results in reduced fertility in mice {Shelly, 2021}. Interestingly, PM alleles show elevated levels of *FMR1* transcription initiation [8]. R-loop formation could potentially contribute to this via its effects on chromatin decondensation [100], inhibition of binding of DNA methyltransferases [101], or the recruitment of activators including the ten-eleven translocation (TET) DNA demethylases [102]. It is also possible that the formation of hairpins or G4 DNA by the non-template strand predisposes these regions to oxidative damage, in turn increasing transcription, as has been described for the *PCNA* gene [103].

Several different models that invoke RNA hairpins formed by CGG-repeats have been proposed to explain PM pathology, as illustrated in Figure 2B. One such model proposes that binding of specific proteins to the CGG-repeat-containing RNA hairpins results in them being sequestered and unable to carry out their normal activities [84,104]. Numbered amongst these proteins are the splicing factor src-associated in mitosis of 68 kDa (Sam68) [104], and the DiGeorge syndrome critical region gene 8 (DGCR8) protein [84], a double-stranded RNA-binding protein involved in the microRNA (miRNA)-processing pathway. Consistent with a role for sequestration of these proteins, Sam68-mediated splicing abnormalities are seen in FXTAS patient cells [104], and decreased levels of mature miRNAs are seen in the brains of FXTAS patients. This is associated with decreased dendritic complexity and reduced viability of neuronal cells in culture that can be reversed by overexpression of DGCR8 [84].

Repeat-associated non-AUG (RAN) translation, a form of translation that initiates at near cognate codons upstream of or within the repeat, has also been suggested to account for PM pathology [85,86,105,106,107], as previously proposed for other REDs [87]. RAN translation is thought to be triggered by the stalling of the ribosome by RNA hairpins, consistent with work suggesting that kinetic barriers to the ribosome favor initiation at otherwise suboptimal initiation codons located upstream of the true initiation codon [108]. In reporter constructs with PM-sized repeat tracts, RAN translation can occur in both the sense strand producing polyglycine (FMRpolyG), polyalanine (FMRpolyA), and polyarginine (FMRpolyR)-containing proteins, and the antisense strand producing polyproline (ASFMRpolyP), polyalanine (ASFMRpolyA), and polyarginine (ASFMRpolyR)-containing proteins. FMRpolyG and FMRpolyA can be seen in intranuclear neuronal inclusions in FXTAS patients using immunochemical detection methods [109,110,111,112], and overexpression of FMRpolyG in particular is toxic in various model systems [86,107].

Interestingly, there are two other potential intersections of RNA structure and protein interactions in RAN translation. The first is related to the fact that many repeat-containing transcripts activate the double-stranded RNA-dependent protein kinase PKR [113,114], presumably due to their ability to form hairpins. This results in an increase in the phosphorylation of eukaryotic translation initiation factor 2 subunit alpha (eIF2α) which in turn exacerbates RAN translation [115]. Supporting the role of PKR in RED pathology is the fact that its inhibition reduces RAN protein expression and improves disease symptoms in a mouse model of *C9orf72* ALS/FTD [114]. Whether PKR plays a similar role in the context of CGG-repeat expansion remains to be seen. The repeats did not cause significant PKR activation in a tissue culture model [36]; however, whether this is due to the cell type used or the level of CGG-RNA produced is unclear. The second intersection with RNA structure is the demonstration that FMRpolyG binds CGG-RNA quadruplex structures in vitro, with evidence of G4 RNA promoting the liquid-to-solid transition and aggregate formation of FMRpolyG in a FXTAS mouse model [45]. However, overexpression of FMRpolyG is not always associated with FXTAS pathology in mice [116]. Furthermore, FMRpolyG is not detected by mass spectroscopy of brain extracts of FXTAS patients [117] and is only present at very low levels in inclusions isolated from such patients [17]. This raises the possibility that despite the immunological detection of these proteins in patient samples, their concentration may be too low to account for the pathology observed in PM carriers.

In addition to PKR activation by the repeat-containing RNA hairpins, elevated type 1 interferon (IFN) signaling is seen in *C9orf72* ALS/FTD [118]. This process, like PKR activation, is part of the normal cellular response to double-stranded RNAs. In ALS/FTD it is associated with sterile inflammation and neuronal death. Cell death can be suppressed by inhibitors of Janus kinase, a key component of the major signaling pathway activated by IFNs but not by PKR inhibitors [118]. Whether a similar effect is seen for the CGG-RNA hairpins in PM carriers remains to be seen.

R-loop formation at the *FMR1* locus has also been proposed as a source of pathology in PM carriers [49,119] as illustrated in Figure 2B. R-loops are prone to single-stranded breaks and DSBs resulting from clustered single-stranded breaks [120]. Hyperphosphorylation of ataxia-telangiectasia mutated kinase (ATM), a consequence of DSBs, is seen in FXTAS cell and animal models, and γH2AX, a marker of double-strand breaks, is present in nuclear inclusions in FXTAS patient tissue [17,97]. However, while mutations that affect R-loop levels genome-wide are associated with a variety of neurodegenerative diseases [121], given the prevalence of R-loops in the genome it is unclear whether the addition of a single, albeit a large and stable, R-loop at a PM allele would be sufficient to trigger neuronal cell death.

In addition to pathology characteristic of PM carriers, many carriers of large PM alleles, or rare FM alleles that do not become silenced, show reduced levels of FMRP that could contribute to some of the symptoms seen in this population [122,123]. The reduced FMRP levels are thought to be due to the stalling of the 40S ribosomal subunit by the hairpin formed by the repeats in the 5′ UTR of the *FMR1* transcript [122,124].

### 4.2. Pathology in FM Carriers

#### 4.2.1. *FMR1* Gene Silencing

The 5′ end of the *FMR1* gene in FM carriers is epigenetically modified, resulting in gene silencing and an absence or deficiency in FMRP. In FM carriers the DNA in this region of the gene is hypermethylated and associated with modified histones typical of heterochromatin, including histone H3 trimethylated at lysine 27 (H3K27Me3) [125]. H3K27Me3 is deposited by the polycomb repressive complex 2 (PRC2). R-loops are important for PRC2-mediated gene silencing at several loci [88]. PRC2 binds to R-loops directly and drives R-loop production in Drosophila [126]. PRC2 has also been reported to bind to G-rich RNA and to G4-forming RNA sequences in particular [127]. R-loops have also been implicated in silencing in both FXS and a related RED, Friedreich ataxia [52]. The *FMR1* transcript is important for recruiting PRC2 to the 5′ end of FM alleles that have been reactivated with 5-deazadeoxycytidine (AZA), a DNA methyltransferase inhibitor [125]. Inhibition of PRC2 or blocking its recruitment to the *FMR1* 5′ UTR prevents H3K27 trimethylation at this locus [50]. This in turn prevents the remethylation and resilencing of FM alleles that typically occur after AZA is withdrawn [50,125]. These data would be consistent with a model in which PRC2 binds to the 5′ end of the *FMR1* transcript, while the transcript is also simultaneously bound to the 5′ end of the *FMR1* gene via an R-loop. This would tether PRC2 in the vicinity of the *FMR1* promoter, as illustrated in Figure 2C. PRC2-mediated H3K27 trimethylation is favored by loss of marks of active chromatin [128,129,130]. This loss could be triggered by R-loop formation itself via increased transcription termination [53,131], or as a downstream consequence of the induction of DNA damage at R-loops [132,133]. Silencing has traditionally been considered to occur when the repeat number exceeds 200 based on data from Southern blotting; however, higher-resolution techniques like capillary electrophoresis suggest that the threshold may be higher than this [134]. What triggers the transition from the hyper-expressed state to the silenced state is unknown and the role of an R-loop in gene silencing of FM alleles seems paradoxical given its proposed roles in hyperexpression of PM alleles. However, there are many reports of similar paradoxical effects of R-loops in the literature (see [135] for a good recent discussion). The R-loop formed by an FM allele while it was still transcriptionally active would be more stable than an R-loop formed on a PM allele. As such the R-loops formed on FM alleles may form a more effective block to transcription elongation. This would result in a larger drop in H3K36me3 levels, which in turn would favor H3K27 trimethylation.

Members of the argonaute protein family and the endoribonuclease DICER1, proteins that are important for RNA-induced gene silencing via the small interfering RNA (siRNA) pathway, have also been suggested to play a role in *FMR1* gene resilencing [136]. This presumably reflects a role for double-stranded RNA in the silencing process. However, whether the source of double-stranded RNA is the RNA hairpin formed by the FX repeats or the product of the annealing of the *FMR1* transcript and an antisense transcript from this locus [137] is unclear. DICER-mediated gene silencing is thought to be accomplished via SUV39H-mediated trimethylation of H3K9 [89]. Since inhibitors of H3K9 methylation [138] and H3K27 trimethylation [50] delay resilencing after AZA treatment, methylation at both residues might be involved in restoring DNA methylation at this locus.

#### 4.2.2. Chromosome Fragility

Fragile sites (FSs) are breaks or gaps that are visible in otherwise condensed chromosomes in metaphase spreads of cells treated with different classes of replication inhibitors [139]. They are thought to represent regions of the genome that are difficult to replicate. In the case of the *FMR1* locus, expression of the fragile site, FRAXA, is induced by folate-stress that causes nucleotide pool imbalances [140]. CGG repeats are known to be difficult to replicate both in vitro [27] and in vivo [141], and replication stalling is seen at the 5′ end of the endogenous *FMR1* gene [142]. Given the ability of CGG-repeat structures to block DNA synthesis in vitro [27], these structures could account for the replication difficulty shown in Figure 2C. The formation of a block to the replication fork is consistent with the fact that FM alleles are prone to mitotic DNA synthesis (MiDAS) when subjected to folate-stress. MiDAS is thought to be a form of break-induced replication (BIR), a salvage pathway involved in the processing of stalled replication forks to allow replication of the chromosome to be completed [90]. Suppression of MiDAS prevents chromosome fragility, but alleles that fail to initiate BIR at all are associated with high levels of ultrafine bridges (UFBs), anaphase bridges involving single-stranded regions of DNA that are histone-free [46]. Failure to resolve these UFBs results in non-disjunction of the chromosomes and subsequent aneuploidy [90] that may account for the high frequency of Turner syndrome observed in female carriers of FM alleles [24].

Replication difficulties may also account for the fact that male PM carriers do not transmit FM alleles to their children since, unlike oocytes which are post-mitotic, male gametes undergo multiple rounds of replication prior to fertilization. As such, there may be selective pressure for smaller alleles in males that is not seen in females.

## 5. Concluding Remarks

While the ability of the FX repeats to form secondary structures of various sorts has been known for some time, work in recent years has begun to identify ways to target these structures or the downstream consequences of these structures, so as to ameliorate their effects. For example, CCG-repeat-containing antisense oligonucleotides (ASOs) reduce R-loop formation and ameliorate some of the downstream consequences of the formation of RNA hairpins [143]. Small molecules that target CGG-RNA hairpins have also been shown to have beneficial effects in cell and mouse models of the PM [144,145,146]. Additionally, the ability of PKR to promote RAN translation can be inhibited by metformin [114], a widely used oral hypoglycemic agent used to treat type 2 diabetes. Thus, an understanding of the secondary structures formed by disease-associated repeats and their downstream consequences is beginning to reveal therapeutic opportunities that may be useful for treating these disorders.
